# Automatic Personal Identification Using a Single MRI Slice

**DOI:** 10.3390/bioengineering13050494

**Published:** 2026-04-24

**Authors:** Andreas Heinrich

**Affiliations:** Department of Radiology, Jena University Hospital, Friedrich Schiller University, Am Klinikum 1, 07747 Jena, Germany; andreas.heinrich@med.uni-jena.de

**Keywords:** computer vision systems, magnetic resonance imaging, human identification, head

## Abstract

Identification of unknown individuals is challenging, and radiological imaging databases provide rich anatomical information for automated recognition. This study evaluated whether a single routine magnetic resonance imaging (MRI) slice contains sufficient person-specific features to identify individuals in large databases. It analyzed 11,078 head MRI examinations from 5770 individuals (age 52 ± 18 years, 2714 men) acquired between 2002 and 2025. For identification, 112 individuals were randomly selected across eight 10-year age groups, and one slice from four anatomical regions was extracted. The remaining 10,966 MRI examinations with 247,804 slices formed the reference database. Distinctive anatomical features were automatically extracted using computer vision (CV), and the identification rate was evaluated by rank. Using a single MRI slice, the identification rate at rank 1 reached 96% (107/112) for the best-performing region, the maxillary sinus, among 5770 potential identities. Across all regions, the rank 1 identification rate ranged from 91% to 96%; combining them increased rank 1 and 10 identification rates to 98% (110/112) and 99% (111/112). Identification rate remained stable over several years, with only two cases showing reduced rank 1 performance, likely due to age-related morphological changes. A single MRI slice contains stable, individualized features sufficient for reliable identification in large databases, supporting automated CV-based personal identification across years.

## 1. Introduction

Identifying unknown individuals remains a major challenge in both forensic and clinical settings [1,2,3,4,5,6,7,8]. Traditional identification methods, such as fingerprints, deoxyribonucleic acid (DNA) analysis, or dental records, require prior reference material and are often unavailable for unknown individuals [9]. When no prior reference or personal clues exist, rapid identification becomes impossible, highlighting a critical gap in current practice. Although radiological imaging databases are widely available, their potential for automated personal identification has not been fully explored. Computer vision (CV) techniques, including classical mathematically interpretable methods as well as newer artificial intelligence (AI)-based approaches, particularly convolutional neural networks (CNNs), can detect, analyze, and interpret visual information. These methods enable automated personal identification by analyzing and comparing radiological images [10]. Prior work has developed CV-based personal identification as a modular approach, combining classical descriptor-based CV for interpretable and robust identification with optional AI-based modules, such as age estimation [11], to improve efficiency. This method has shown a high identification rate in modalities including panoramic radiographs (PRs) [11] and computed tomography (CT) [12,13,14,15]. However, its applicability to routine head magnetic resonance imaging (MRI) remains largely unexplored, leaving unanswered questions about which anatomical CV features are most informative and whether single slices suffice for identification. Previous MRI studies have focused exclusively on the brain [16,17,18] and facial structures [19], leaving other head regions untested. Certain head structures, including the frontal and maxillary sinuses, exhibit person-specific morphology [20,21,22,23,24,25,26,27], making them suitable targets for automated identification.

This study aimed to evaluate whether a single routine head MRI slice contains sufficient individualized anatomical information to enable reliable automated CV-based personal identification within a large clinical imaging database.

## 2. Materials and Methods

The study was approved by the local institutional review board (IRB) at Jena University Hospital (registration number 2019-1505-MV). Due to the retrospective nature of the investigation, written informed consent was waived by the IRB at Jena University Hospital. All methods were performed in accordance with the relevant guidelines and regulations, and the study was conducted in accordance with the Declaration of Helsinki.

### 2.1. Study Population and Image Acquisition

A total of 11,078 head MRI examinations from 5770 individuals (age 52 ± 18 years, range 1–92 years; 2526 female, 2714 male, 530 unknown) acquired between November 2002 and March 2025 were included. Only T1-weighted turbo spin-echo sequences labeled “t1_se_tra” in the Picture Archiving and Communication System (PACS) were eligible. Examinations were acquired on 15 clinical scanners (TR 465.7 ± 62 ms, TE 8.9 ± 1.7 ms, slice thickness 5.2 ± 0.7 mm, in-plane voxel size 0.55 ± 0.14 mm) from a single vendor (Siemens Healthineers, Erlangen, Germany), predominantly at 1.5 T, with a smaller number at 1 T or 3 T.

### 2.2. CV Feature Extraction and Matching Process

Distinctive local anatomical structures were automatically detected as keypoints and encoded as numerical descriptors using a CV-based personal identification framework adapted from prior PR and CT studies [11,12,13,14]. The CV pipeline was implemented in C++ using OpenCV 4.12.0 [28]. The algorithmic framework and parameter optimization are described in detail in previous studies.

**Figure 1:**
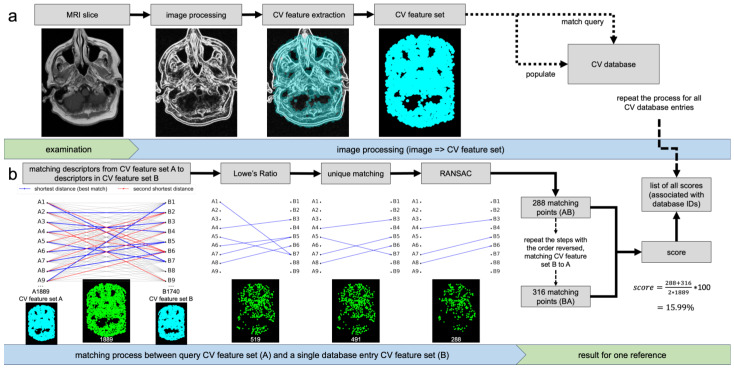
Overview of the CV-based personal identification pipeline. (**a**) Feature extraction from preprocessed MRI slices. (**b**) Descriptor matching and score computation between query and reference CV feature sets.

Figure 1a illustrates the automated CV feature extraction pipeline. Each MRI slice undergoes a standardized preprocessing workflow including intensity normalization to 8-bit grayscale, edge enhancement, and noise reduction. Edge enhancement was performed using multi-directional Sobel filters (eight orientations from 0° to 315°). The Sobel gradient parameter was increased to 1.8 to enhance boundary contrast. The directional responses were fused using a maximum-intensity strategy to generate a single edge-enhanced image. Subsequently, noise was reduced using an averaging filter with a fixed kernel size to suppress high-frequency variations while preserving relevant structural information. Following preprocessing, CV features were automatically detected using the AKAZE algorithm [29,30], which identifies stable local image structures and represents them as scale- and rotation-invariant keypoints with associated descriptors. A previously optimized parameter set [12,13] was applied, including a Sobel gradient of 1.8, an averaging filter size of 3, 4 octaves and layers, diffusivity PM_G2, the DESCRIPTOR_KAZE configuration, and a detection threshold of 0.001. The resulting CV feature sets are decoupled from the source image and stored in a CV database with associated metadata (e.g., patient ID) or used as query features for matching.

As illustrated in Figure 1b, matching is performed by comparing CV feature descriptors between query and reference slices. For each query descriptor, similarity to all reference descriptors is computed using squared Euclidean distance, appropriate for floating-point KAZE descriptors. The two nearest neighbors are identified, and Lowe’s ratio test (threshold 0.6) is applied to retain only reliable correspondences by rejecting ambiguous matches. A unique matching constraint is then enforced to ensure one-to-one correspondences between query and reference descriptors. To enhance robustness, Random Sample Consensus (RANSAC)-based homography estimation is used to filter outliers and ensure geometric consistency, employing a reprojection threshold of 2. The number of validated matches is used to compute a similarity score defined as the ratio of matching points to the total number of CV features in the query slice, multiplied by 100 and expressed as a percentage ranging from 0% (no matches) to 100% (all features matched). To reduce directional bias, matching is performed in both directions (query-to-reference and reference-to-query), and the final similarity score is obtained as the mean of both values. The resulting similarity scores for all database entries are stored together with their corresponding identifiers, enabling ranking-based identification.

### 2.3. Evaluation

**Figure 2:**
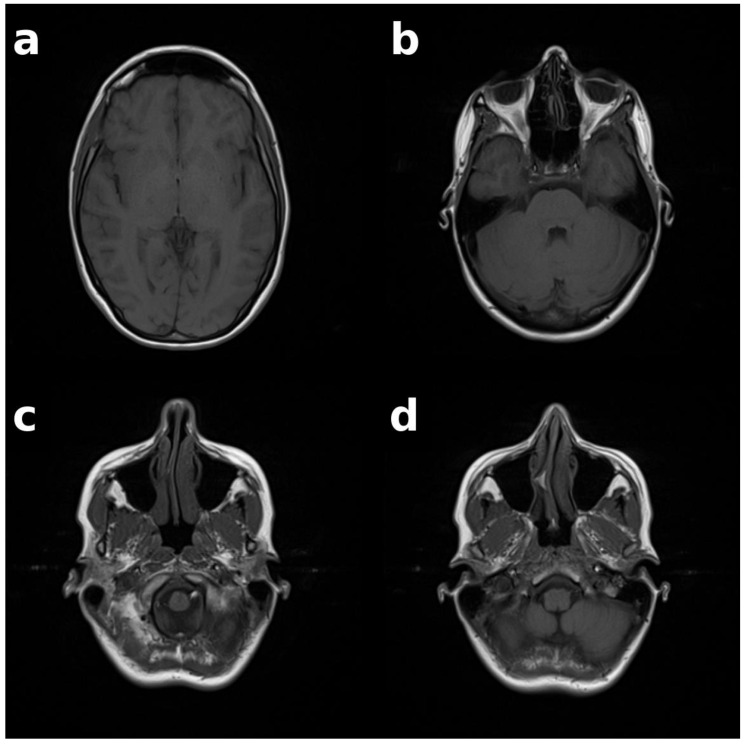
Query MRI slices from one individual. Four anatomical regions are illustrated as single representative 2D cross-sections: (**a**) frontal sinus, (**b**) ethmoid bone, (**c**) nasal septum, (**d**) maxillary sinus.

For the identification experiments, 112 individuals (56 female, 56 male) were randomly selected with equal distribution across eight 10-year age groups (10–89 years). For each participant, the most recent examination was chosen, and a single slice was extracted from each of four predefined anatomical regions (see Figure 2): frontal sinus, ethmoid bone, nasal septum, and maxillary sinus, resulting in 448 query slices. Slice selection was performed by identifying a representative slice within each anatomical region. If multiple slices met this criterion, the slice closest to the anatomical center of the respective structure was selected. This approach aimed to obtain a 2D cross-section representative of the underlying 3D anatomy, acknowledging that anatomical structures are only partially captured in a single slice.

**Figure 3:**
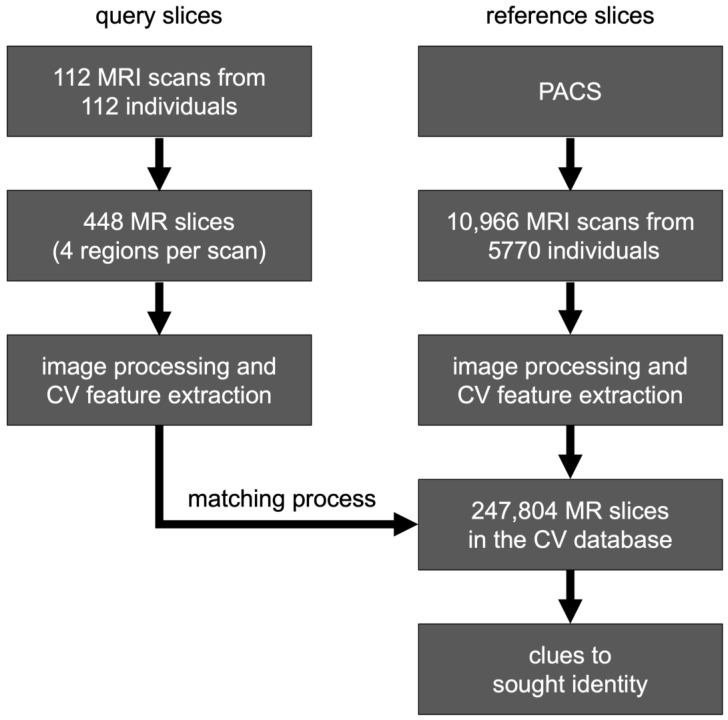
Workflow of CV-based personal identification. From 5770 individuals and 11,078 head MRI examinations, 112 were randomly selected (most recent MRI as test cases, 448 query slices across four regions) and matched against 247,804 reference slices from the remaining 10,966 examinations; no reference slice was acquired on the same day as its corresponding query slice. Cases were balanced across age decades (10–89 years) and sex (male/female: 224/224).

All remaining MRI examinations were included in the reference CV database, comprising 247,804 slices from 10,966 MRI series of 5770 individuals (see Figure 3 and Table 1). MRI series acquired on the same day as the query slices from the same individuals were explicitly excluded from the CV database and are not included in these counts.

For each query slice, matching was performed against all entries in the reference CV database, and similarity scores were stored together with the corresponding identifiers. For each individual, the maximum similarity score across all associated database entries was used to obtain a person-level decision, and a global ranking of all individuals in the database was generated.

Identification performance was evaluated using rank-based metrics. A correct rank 1 identification was defined as the correct individual appearing at the top of the ranked list, while rank 5 and rank 10 accuracies were defined as inclusion of the correct individual within the top 5 or top 10 identities, respectively. Ties in similarity scores were resolved conservatively by assigning the worst-case rank to all tied identities. This approach reflects ambiguity in cases where individuals cannot be distinguished due to identical similarity scores.

**Figure A1:**
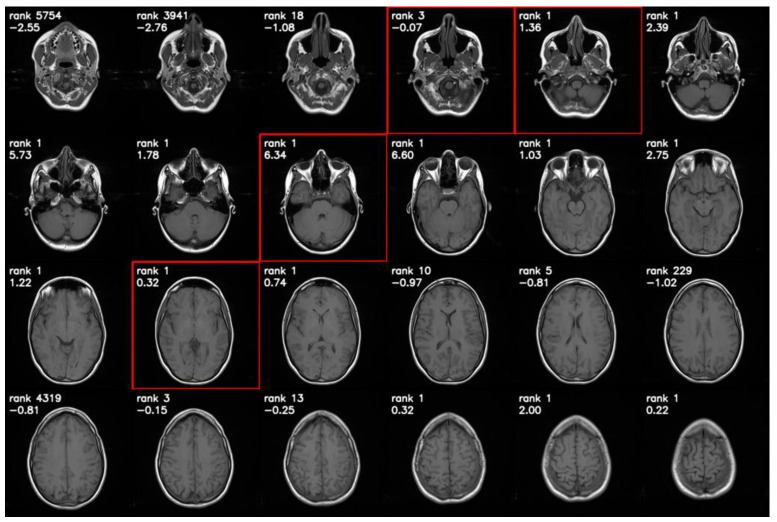
Full MRI slice series of one examination with highlighted evaluation slices (indicated by red boxes) used in this study. Each slice is annotated with its rank-based identification result based on matching against 247,804 slices from 10,966 MRI series of 5770 individuals; ties in similarity scores were resolved conservatively by assigning the worst-case rank. Additionally, the score difference between the correct identity and the best competing identity is shown below the rank annotation. The reference series of the same individual begins at a higher cranial level, which explains the higher ranks observed in the first slices. The example demonstrates robust rank 1 identification across multiple adjacent slices within the same anatomical region.

To illustrate identification behavior across an entire examination, all slices of one representative MRI series were matched against the full CV database, rather than limiting the analysis to the four predefined regions (see Figure A1). Robustness under anonymization preprocessing was further assessed by applying defacing and skull-stripping [31], and re-evaluating the same examination using a reduced reference set. For each of the 24 query slices, this reduced set consisted of the target subject and the 20 highest-scoring reference individuals for each slice, obtained from the full CV database matching shown in Figure A1.

Statistical uncertainty was quantified by computing 95% confidence intervals (CIs) for identification rates using the Wilson score method for binomial proportions, treating each query outcome as a binary event (correct vs. incorrect identification at the respective rank threshold). Differences in rank 1 identification performance between anatomical regions were assessed using Cochran’s Q test for related proportions. Post hoc pairwise comparisons were performed using exact McNemar tests with Holm–Bonferroni correction for multiple comparisons. Statistical significance was defined as *p* < 0.05.

## 3. Results

**Figure 4:**
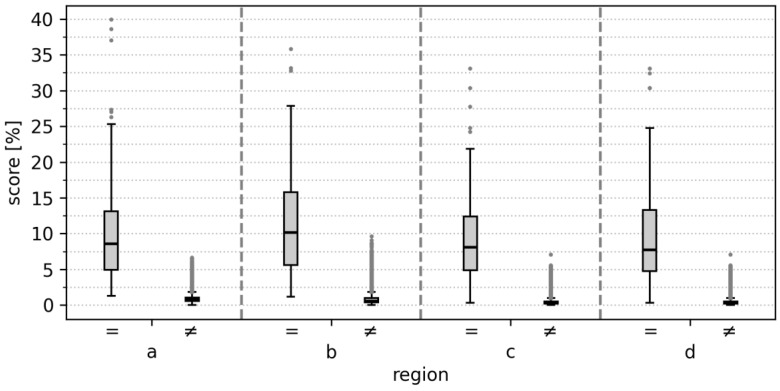
Boxplots of CV similarity scores for same (=) vs. different (≠) individuals across four regions: (**a**) frontal sinus, (**b**) ethmoid bone, (**c**) nasal septum, (**d**) maxillary sinus.

Using a single MRI slice, 96% (107/112) of individuals were correctly identified at rank 1 among 5770 potential identities, with the maxillary sinus (region d) showing the highest rank 1 identification rate. Identification performance was consistently high across all four predefined anatomical regions, with the rank 1 identification rate ranging from 91% to 96%, rank 5 from 92% to 97%, and rank 10 from 93% to 97% (see Table 2). The mean score for comparisons of same-individual images was 10.41 ± 1.00%, compared to 0.62 ± 0.22% for different individuals (see Figure 4). Median scores and identification rates stratified by age are presented in Table 3, showing no apparent systematic differences across decades.

No statistically significant differences in rank 1 identification performance were observed between anatomical regions (Cochran’s Q test: Q = 4.12, *p* = 0.249), indicating no evidence of heterogeneity across regions. Post hoc pairwise McNemar tests with Holm–Bonferroni correction confirmed the absence of significant differences between any region pairs (all adjusted *p* ≥ 0.375).

When combining information from all four regions, rank 1 identification increased to 98% (110/112) and rank 10 to 99% (111/112). Only two cases were not identified at rank 1: one adolescent (best rank 137) with a 7-year interval between MRI examinations (age 10 vs. 17 years), and one adult (best rank 6) with a 5-year interval (age 31 vs. 36 years), likely reflecting age-related morphological changes.

**Figure 5:**
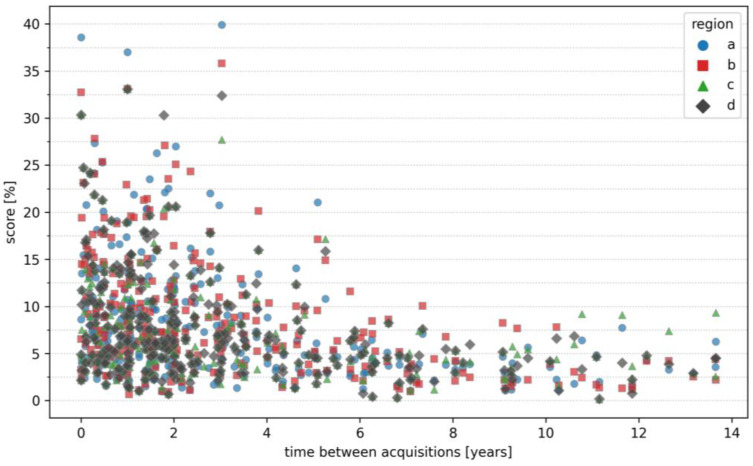
Relationship between score and time between MRI acquisitions of the same individual across four regions: (a) frontal sinus, (b) ethmoid bone, (c) nasal septum, (d) maxillary sinus.

**Figure 6:**
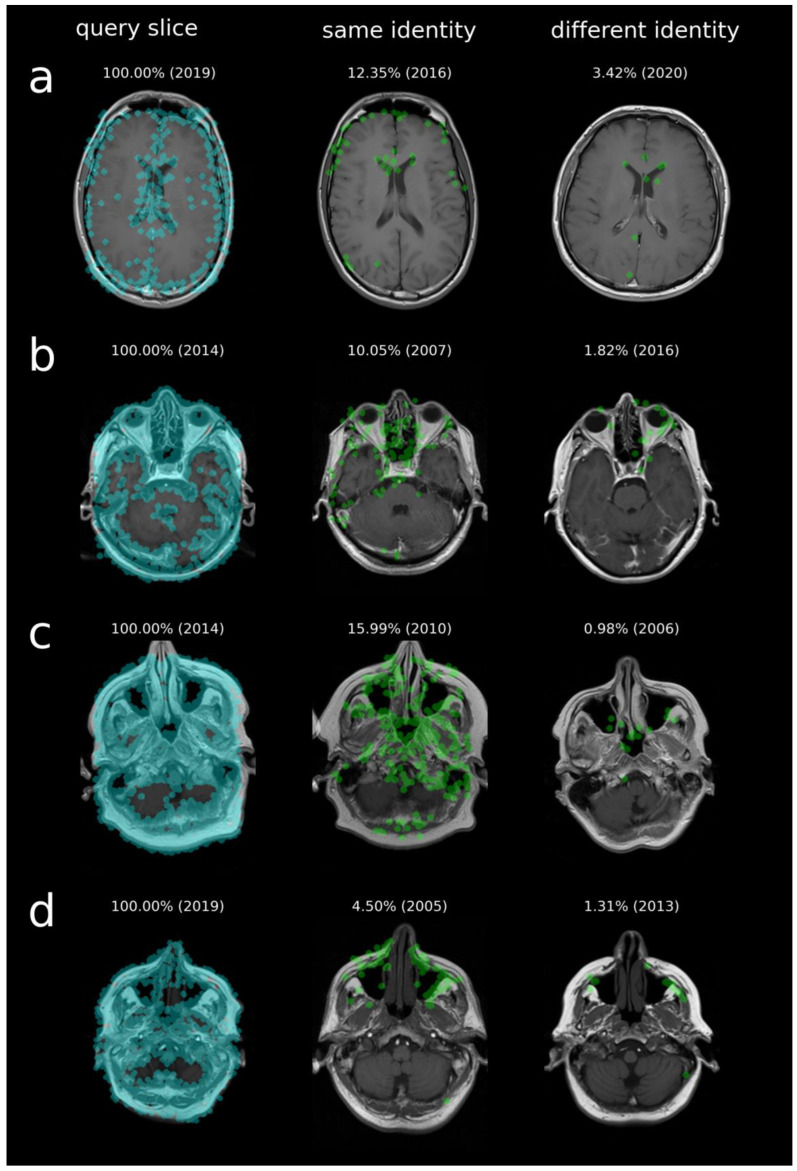
Representative examples of successful identification using individualized anatomical CV features in MRI slices: (**a**) frontal sinus, (**b**) ethmoid bone, (**c**) nasal septum, (**d**) maxillary sinus. (**Left**): query slice with detected CV features (blue). (**Middle**): reference slice from the same individual; (**right**): best-matching slice from a different individual. Matched CV feature points are shown in green. Despite some anatomical similarities across individuals, the correct reference image is clearly distinguishable, enabling reliable identification. Acquisition year and similarity score (%) are shown.

Identification rate remained robust even for slices acquired several years apart (up to 14 years), indicating stability of individualized anatomical CV features (see Figure 5). Rank 1 identification was also successful across different MRI protocols and scanner types. Among the correctly identified rank 1 cases, the mean absolute differences were 25.79 ± 53.13 ms for TR, 0.08 ± 0.24 ms for TE, and 0.17 ± 0.42 mm for slice thickness (maximum absolute differences: 376 ms, 1 ms, and 2 mm, respectively). A systematic subgroup analysis was not feasible due to the limited number of examinations per specific configuration. In Figure 6, an example of identified matching points is shown. CV features are automatically detected in the query slice (blue dots) and are matched in the reference slice (green dots). While some anatomical similarities can lead to matches between different individuals, these scores are generally much lower than those observed for the same individual.

**Figure 7:**
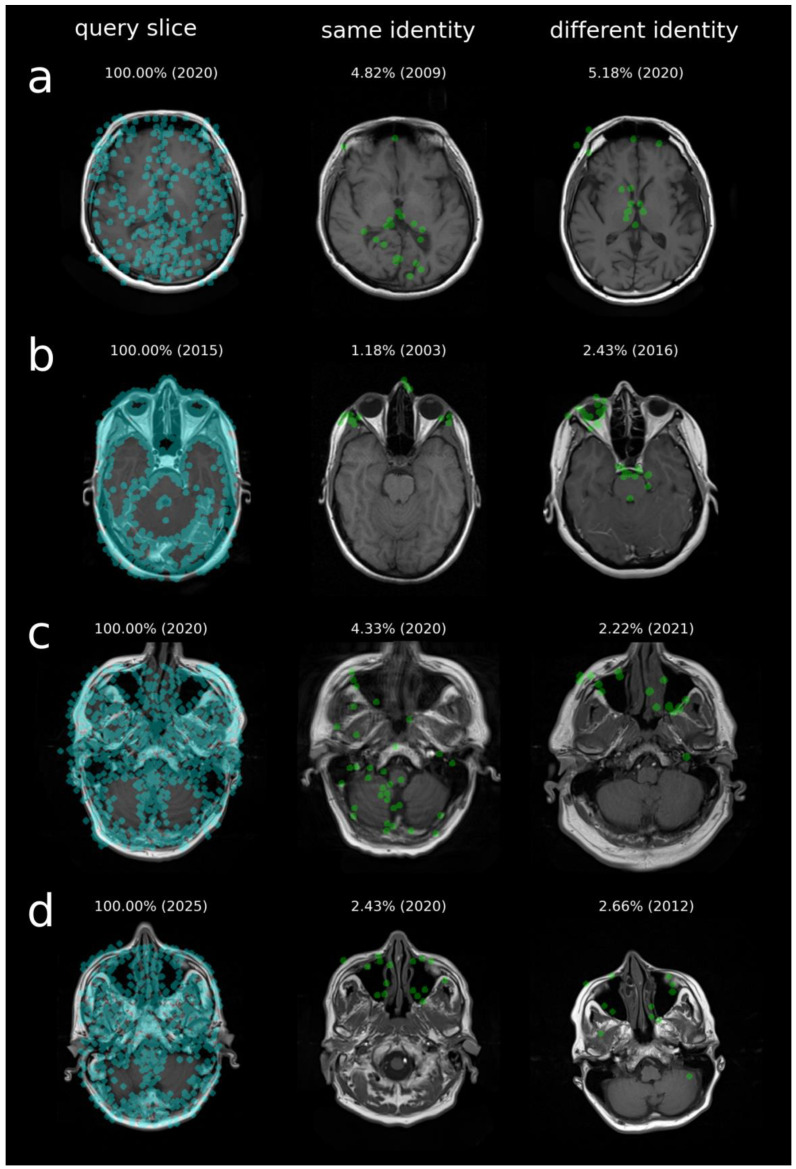
Examples of cases with reduced identification performance. (**Left**): query slice with detected CV features (blue). (**Middle**): reference slice from the same individual; (**right**): best-matching slice from a different individual. Matched CV feature points are shown in green. Reduced matching performance may occur with long time intervals between examinations (**a**,**b**), motion artifacts (**c**), or more pronounced anatomical differences due to variations in head orientation or positioning between query and reference images (**d**). Acquisition year and similarity score (%) are shown.

Reduced matching performance can occur due to susceptibility or motion artifacts in the reference image, differences in head orientation between query and reference slices, or anatomical changes over long intervals between scans. Figure 7 illustrates scenarios in which these factors can reduce the number of matched keypoints, highlighting how such variations affect identification performance.

**Figure A2:**
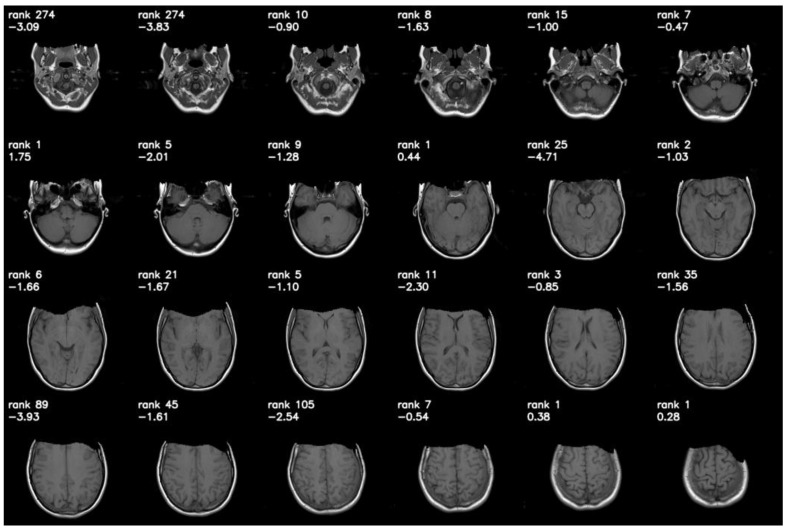
Full MRI slice series of one examination after defacing. Each query slice was matched against a reduced reference set of 474 slices from 274 individuals, obtained by selecting the slices of the 20 highest-scoring identities from the results of Figure A1 and merging the results across all queries. Each slice is annotated with its rank-based identification result and the score difference between the correct identity and the best competing match.

**Figure A3:**
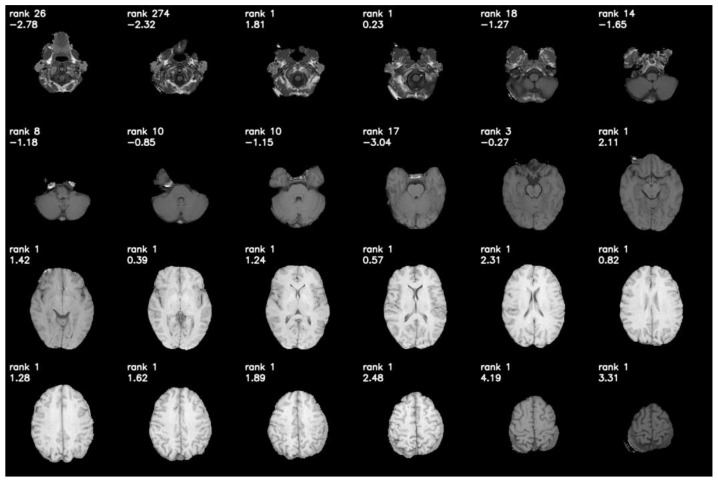
Full MRI slice series of one examination after skull stripping. Each query slice was matched against a reduced reference set of 474 slices from 274 individuals, obtained by selecting the slices of the 20 highest-scoring identities from the results of Figure A1 and merging the results across all queries. Each slice is annotated with its rank-based identification result and the score difference between the correct identity and the best competing match.

**Figure A4:**
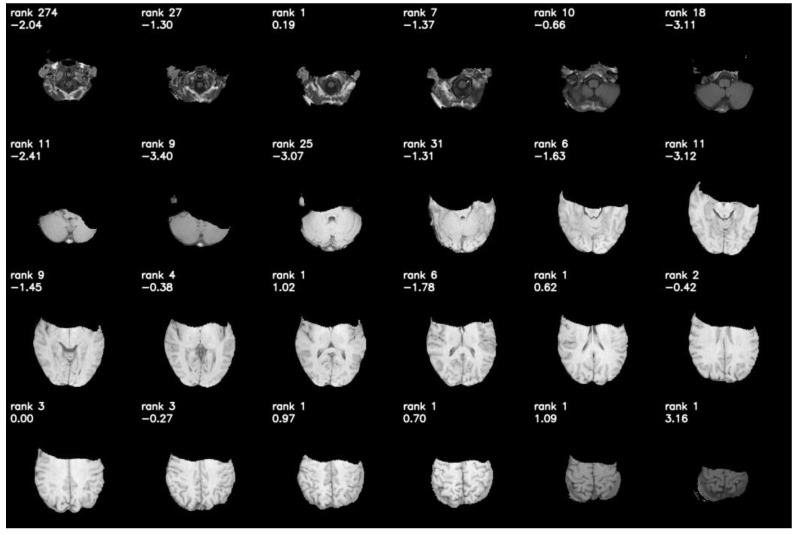
Full MRI slice series of one examination after defacing and skull stripping. Each query slice was matched against a reduced reference set of 474 slices from 274 individuals, obtained by selecting the slices of the 20 highest-scoring identities from the results of Figure A1 and merging the results across all queries. Each slice is annotated with its rank-based identification result and the score difference between the correct identity and the best competing match.

Rank 1 matches were still achievable after defacing (see Figure A2), skull stripping (see Figure A3), and combined preprocessing (see Figure A4), across multiple slices.

## 4. Discussion

This study demonstrates that even a single routine MRI slice contains sufficient individualized anatomical information to reliably identify individuals within a large clinical imaging database. Using CV-based personal identification, the rank 1 identification rate exceeded 96% among 5770 potential identities, with robust performance across multiple anatomical regions and over several years between acquisitions. Among the analyzed regions, the nasal septum and maxillary sinus showed the highest discriminative performance, likely reflecting their highly individual and structurally stable morphology.

These findings complement previous work demonstrating successful CV-based personal identification across several radiological modalities, including PRs [11], head CT [12,13], body CT [14], and cross-modal CT-to-PR comparisons [15]. Together, these studies indicate that both planar and volumetric imaging modalities contain stable anatomical signatures that enable reliable recognition of individuals. The present study extends this concept to MRI and shows that the same CV-based framework can be applied to a fundamentally different imaging modality. Furthermore, in analogy to CT-to-PR identification [15], an automated MRI-to-PR approach is conceivable and could further expand the availability of reference data. While MRI data can already be used to reconstruct PR-like images [32,33], these methods currently rely on manual processing, and the presence of metallic implants or fillings can induce artifacts that degrade image quality [34]. From a technical perspective, routine MRI acquisition appears to facilitate robust slice-to-slice matching. Multi-millimeter slices provide tolerance to minor variations in head positioning, reducing the impact of small changes on structures outside the target region. In contrast, thinner slices, as often used in CT, are more sensitive to head orientation differences, which can complicate direct slice-to-slice comparisons [12,13]. This likely contributed to the high identification performance observed in the present study. Although four representative slices per examination were selected for standardized evaluation, this applied only to the query data; the reference CV database comprised the complete set of MRI series from all reference examinations without region-specific selection. As illustrated in Figure A1, each anatomical region spans multiple adjacent slices, and the framework can be applied automatically to all query slices without manual selection. For each query slice, matching against the CV database yields a rank-based result, and the identity of the unknown individual can be determined based on the highest similarity score or the margin between the best and second-best match. Various alternative aggregation strategies are also possible, including combined evaluation across query slices; however, these were not the focus of the present work. Overall, exact slice selection is not essential for reliable identification, as correct rank 1 matches are consistently observed across a broad range of adjacent slices. In addition, the dataset comprised 15 scanners with varying field strengths and slice thicknesses. Although a systematic subgroup analysis was not feasible because only a limited number of examinations was available per configuration, the consistently high identification performance across many years suggests robustness to protocol- and hardware-related variability. This is further supported by the scale-invariant nature of AKAZE, which can detect stable local anatomical features despite minor differences in resolution or acquisition parameters.

Previous MRI-based identification studies [16,17,18] have primarily focused on brain morphology. Takao et al. [16] demonstrated that voxel-based morphometry combined with principal component analysis allowed near-perfect identification of 215 individuals scanned over a one-year interval. Similarly, Ali and Ali [17] reported identification accuracies of 91–93% using machine-learning approaches applied to brain MRI images. Wachinger et al. [18] introduced the BrainPrint framework, which represents cortical and subcortical structures using shape descriptors derived from the Laplace-Beltrami spectrum and achieved identification accuracies above 99.8% in a longitudinal dataset of approximately 700 subjects. In addition, Steeg et al. [19] showed that three-dimensional renderings of MRI head images could be re-identified using publicly available facial recognition software in four tested cases, with successful identification in 50%, highlighting potential privacy risks associated with imaging data. In contrast to these studies, which focus primarily on brain morphology or facial reconstruction, the present work demonstrates that reliable identification can also be achieved using non-brain anatomical structures from a single routine MRI slice. Structures such as the nasal septum or paranasal sinuses exhibit considerable inter-individual variability while remaining relatively stable over time, making them suitable anatomical targets for automated identification.

Many AI-based identification methods, particularly those developed for PR and CT, use CNNs to extract features from the entire image and encode them as high-dimensional, global feature vectors [10]. Identification is then performed by comparing these global representations [35,36,37,38,39,40,41,42,43]. While powerful in controlled settings, these vectors are sensitive to temporal or localized anatomical changes (growth, accidents, surgical procedures, tooth loss, restorations, implants), because even small alterations in the image can substantially change the global feature vector, reducing comparability for images of the same individual across time points. They also rely heavily on the quantity, quality, and representativeness of the training data, which can make longitudinal matching of the same individual and transfer to heterogeneous clinical datasets challenging. In contrast, the CV-based personal identification approach applied in this study relies on local keypoints and their descriptors, offering several structural advantages:

1. Training-free operation. No annotated training data are required, eliminating the need for large labeled datasets and avoiding training-induced biases. This also allows the method to be applied across different modalities and imaging protocols.
2. Local, interpretable CV feature matching. Instead of comparing global image representations, the method matches distinctive local keypoints. Successful identification requires only a sufficient number of stable keypoints, so temporal or localized anatomical changes do not necessarily degrade performance. The matched keypoints are readily interpretable, allowing correspondences to be verified transparently.
3. Selective masking of irrelevant regions. Uninformative or potentially confounding areas can be masked without affecting identification performance, providing a practical advantage over global CNN-based approaches.

These conceptual differences do not imply that AI-based approaches are unsuitable for identification tasks. Rather, they highlight that deterministic descriptor-based methods provide complementary advantages, particularly regarding interpretability, scalability, and independence from training data. This motivates a modular design in which descriptor-based matching forms the interpretable, training-free core, while AI components can serve a complementary role by estimating attributes such as age [11,44], sex [45,46], or weight [10,47,48,49] from the query images. This allows the CV database to be filtered in advance, reducing the number of individuals that need to be compared and increasing efficiency.

Fundamentally, CV-based personal identification should be understood as a database-matching approach that helps locate suitable reference material and generate clues about the sought individual. By narrowing the pool of potential individuals from thousands of identities, it can support subsequent legally robust forensic identification based on appropriate reference material and expert review. The proposed framework requires at least one prior examination of the same individual to be present in the CV database. Performance may decrease in the presence of pronounced anatomical changes, long inter-scan intervals, or reduced image quality, as these factors can reduce the number of stable matching points and make the sought individual harder to distinguish from other identities. Accordingly, high ranks may sometimes fail to yield a useful lead even when the correct individual is contained in the database.

Another important consideration is data protection. The CV database does not store the original images; instead, it contains abstract numerical descriptors representing local keypoints. These descriptors are information-reduced local image features used for matching and are associated with a dataset identifier. While previous studies have shown that certain feature representations can leak visual information, including local binary descriptors such as Binary Robust Independent Elementary Features (BRIEF) and Fast Retina Keypoint (FREAK) under strong inverse-problem assumptions [50], and Scale-Invariant Feature Transform (SIFT) features in both classical [51] and deep learning-based reverse attacks [52,53,54], these approaches are typically based on natural image datasets rather than medical imaging data. They also generally rely on additional spatial information, external priors, or strong dataset-specific assumptions. In particular, reconstruction quality is limited, especially when keypoint coordinates are unavailable, as descriptors alone do not explicitly encode the spatial arrangement of image content and still require additional assumptions for reconstruction [53]. Furthermore, it remains unclear to what extent reconstruction approaches developed for natural images can be transferred to medical imaging data, which differ substantially in texture characteristics, noise properties, and anatomical structure. To the best of current knowledge, no comparable reconstruction study exists for AKAZE descriptors. Unlike SIFT, AKAZE utilizes a non-linear diffusion filtering framework to build the scale space, which preserves object boundaries and complicates straightforward mathematical inversion. Moreover, in the present study, AKAZE features are extracted from a preprocessed representation of the MRI slice (see Figure 1). Consequently, even a hypothetical inversion of the descriptors would, at best, reconstruct the preprocessed image representation generated prior to feature extraction, rather than the original MRI slice. Crucially, any such reconstruction would be expected to lack the intensity fidelity and fine-grained anatomical detail required for clinical interpretation or reliable identification beyond the specific matching task, thereby limiting its practical relevance for privacy breaches involving patient health data. Furthermore, feature matching is primarily descriptor-based, whereas keypoint coordinates are used only in a subsequent geometric verification stage based on RANSAC. This improves the result but is not strictly required for matching itself, which means that a CV database can, in principle, rely solely on stored descriptors for the matching process. This design supports a privacy-preserving federated identification setting, as sensitive imaging data remain within the originating institution while matching is performed solely via descriptors associated with a dataset identifier (e.g., indicating the data origin or a pseudonymized patient identifier). Even after standard de-identification procedures such as defacing or skull-stripping [31], many stable anatomical structures are likely to remain detectable and could potentially be matched using CV-based personal identification (see Figure A2, Figure A3 and Figure A4), suggesting that the method may be robust to common anonymization steps. These considerations highlight the need to balance identification performance with potential privacy implications in real-world applications.

Beyond technical feasibility, the present findings raise important ethical and data protection considerations. The demonstrated capability for identification from routine MRI data implies that even de-identified imaging datasets may contain stable biometric information, depending on database size, access conditions, and availability of reference data. The proposed approach is therefore a dual-use technology, with applications in forensic identification, disaster victim identification, and clinical data linkage, where rapid and reliable identification of unknown individuals is valuable. Consequently, deployment in real-world settings should follow strict governance frameworks, including access control, data minimization, pseudonymization, and purpose limitation. Although feature-based descriptors avoid storing raw image data, they still retain sufficient discriminatory information to enable reliable identification when adequate reference data are available.

Several limitations of this study should be acknowledged. Successful identification depends on the presence of reference examinations of the same individual in the CV database, as automated matching is not possible without prior images. The present evaluation is therefore based on a closed-set design, in which all query individuals are included in the CV database. While this enables controlled assessment of ranking performance, it may overestimate performance in open-set real-world scenarios. The number of stable matching CV features can be reduced by both long-term anatomical changes, including growth, surgical interventions, trauma, or disease-related remodeling, and technical variabilities, such as different MRI protocols, reconstruction parameters, or susceptibility artifacts [34]. The present analysis focused on T1-weighted transverse slices from predefined anatomical regions, so performance with other sequences or regions remains to be evaluated in future studies. Moreover, the study was conducted at a single institution, and no external validation on a separate dataset from a different institution, scanner vendor, or population was performed; therefore, generalizability across centers and imaging protocols cannot yet be fully established and requires future multicenter validation.

## 5. Conclusions

In conclusion, a single routine MRI slice contains sufficient individualized anatomical information for reliable identification within large clinical imaging databases. These results demonstrate that CV-based personal identification can be successfully applied to MRI without modality-specific algorithmic modifications. They extend previous work in radiological identification and support the concept of modality-independent imaging-based identification systems that may enhance clinical workflows, forensic investigations, and disaster victim identification, while maintaining transparency, interpretability, and high standards of data protection.

## Figures and Tables

**Table 1:** Demographics of study participants included in the CV database, which served as the reference for the identification process.

Age [Years]	CV Database			
	All	Female	Male	Unknown
1–9	125	60	51	14
10–19	390	185	166	39
20–29	845	396	398	51
30–39	1185	473	638	74
40–49	1878	841	832	205
50–59	2242	1028	980	234
60–69	2277	1038	1069	170
70–79	1643	680	843	120
80–89	376	160	171	45
90–92	5	1	3	1
all	10,966	4862	5151	953

**Table 2:** CV-based personal identification results.

Region	Score [%]				MED Ratio	Identification Rate [%], 95% CI		
	MW (=)	MED (=)	MW (≠)	MED (≠)		Rank 1	Rank 5	Rank 10
a	10.69 ± 7.94	8.60	0.86 ± 0.46	0.74	11.62	91.07 [84.3, 95.1]	91.96 [85.4, 95.7]	92.86 [86.5, 96.3]
b	11.69 ± 7.53	10.12	0.76 ± 0.59	0.59	17.15	92.86 [86.5, 96.3]	94.64 [88.8, 97.5]	94.64 [88.8, 97.5]
c	9.45 ± 6.43	8.09	0.42 ± 0.37	0.32	25.28	94.64 [88.8, 97.5]	97.32 [92.4, 99.1]	97.32 [92.4, 99.1]
d	9.80 ± 7.01	7.74	0.44 ± 0.39	0.34	22.76	95.54 [90.0, 98.1]	96.43 [91.2, 98.6]	97.32 [92.4, 99.1]
comb.	10.41 ± 1.00	8.64	0.62 ± 0.22	0.50	17.28	98.21 [93.7, 99.5]	98.21 [93.7, 99.5]	99.11 [95.1, 99.8]

Mean (MW) and median (MED) scores are shown for same (=) vs. different (≠) individuals. The MED ratio is defined as the median score for the same individual divided by the median score for different individuals. For each query slice, only the highest similarity score for each individual was used, resulting in 112 scores for same-individual matches and 646,128 scores for different-individual matches per region. Identification rates are reported with 95% confidence intervals (CIs). Four regions: (a) frontal sinus, (b) ethmoid bone, (c) nasal septum, (d) maxillary sinus.

**Table 3:** CV-based personal identification results by age group.

Age [Years]	Score [%]		Identification Rate [%]	
	MED (=)	MED (≠)	Rank 1	Rank 10
(a) frontal sinus				
10–19	6.69	0.81	85.71	85.71
20–29	7.10	0.82	78.57	78.57
30–39	9.88	0.80	92.86	92.86
40–49	8.83	0.71	100	100
50–59	7.84	0.70	100	100
60–69	9.70	0.75	78.57	92.86
70–79	12.23	0.65	100	100
80–89	6.80	0.76	92.86	92.86
(b) ethmoid bone				
10–19	7.34	0.69	92.86	92.86
20–29	13.19	0.53	85.71	85.71
30–39	10.54	0.63	85.71	85.71
40–49	9.69	0.52	85.71	100
50–59	9.63	0.60	100	100
60–69	9.62	0.63	100	100
70–79	14.65	0.53	100	100
80–89	7.27	0.64	92.86	92.86
(c) nasal septum				
10–19	4.90	0.36	92.86	92.86
20–29	9.39	0.34	100	100
30–39	8.32	0.35	85.71	92.86
40–49	8.55	0.30	100	100
50–59	7.74	0.31	100	100
60–69	7.75	0.32	85.71	92.86
70–79	9.29	0.29	100	100
80–89	5.11	0.33	92.86	100
(d) maxillary sinus				
10–19	4.71	0.36	92.86	92.86
20–29	9.39	0.36	92.86	92.86
30–39	7.91	0.36	92.86	100
40–49	7.28	0.32	100	100
50–59	7.53	0.31	100	100
60–69	7.88	0.33	85.71	92.86
70–79	11.81	0.30	100	100
80–89	5.54	0.35	100	100

Median (MED) scores are shown for same (=) vs. different (≠) individuals. Each age group of a region includes 7 female and 7 male individuals.

## Data Availability

The datasets analyzed during the current study are not publicly available due to privacy concerns. However, all relevant data supporting the findings of this study are included in the article. For further inquiries or data requests, please contact the corresponding author.

## Abbreviations

The following abbreviations are used in this manuscript:

AI	Artificial Intelligence
CNN	Convolutional Neural Network
CT	Computed Tomography
CV	Computer Vision
DNA	Deoxyribonucleic acid
MRI	Magnetic Resonance Imaging
PACS	Picture Archiving and Communication System
RANSAC	Random Sample Consensus
PR	Panoramic Radiograph
